# High-carbon bio-organic fertilizer reshapes soil carbon fractions and aggregate structure while maintaining high carbon stocks in red soil

**DOI:** 10.3389/fmicb.2026.1775969

**Published:** 2026-03-20

**Authors:** Xiangtong Shen, Yalong Kang, Yongcheng Wang, Jiangfei Gao, Xun Li, Fengchao Liu, Qiong Li, Yuquan Wei, Hongguang Cai, Yuyun Wang, Zhi Xu

**Affiliations:** 1 International Joint Laboratory for Resource Utilization of Agricultural Solid Waste in Yunnan Province, College of Resources and Environmental Science, Yunnan Agricultural University, Kunming, China; 2 College of Resources and Environmental Science, China Agricultural University, Beijing, China; 3 Institute of Agricultural Resources and Environmental Science, Jilin Academy of Agricultural Sciences, Changchun, China

**Keywords:** carbon storage, high-carbon bio-organic fertilizer, microbial communities, soil organic carbon fractions, soil aggregate

## Abstract

**Introduction:**

The application of bio-organic fertilizers is key to enhancing soil carbon storage, but their carbon inputs often fall short of microbial demands, limiting their long-term impact. High-carbon bio-organic fertilizers, which contain more than 40% organic matter, are used to improve soil fertility, but their effects on soil aggregate stability, organic carbon fractions, and bacterial communities, and the consequent impacts on soil carbon storage, are not well understood.

**Methods:**

This study employed a field experiment with four treatments: chemical fertilizer alone (control, CK), organic fertilizer (OF), bio-organic fertilizer (BOF), and high-carbon bio-organic fertilizer (HBOF).

**Results:**

The results showed that organic fertilizer treatments significantly increased organic carbon content across all aggregate size classes, promoted the formation of large aggregates, and improved aggregate stability compared with the CK treatment. The HBOF treatment increased the proportion of soil aggregates larger than 2 mm by 8.58% and 6.87% compared with the OF and BOF treatments, respectively. All organic fertilizers boosted soil carbon storage and active carbon fractions, particularly particulate organic carbon (POC). Under HBOF, POC and microbial biomass carbon (MBC) in active carbon fractions increased by 15.83% and 24.05%, respectively, compared to BOF. The HBOF treatment reduced bacterial diversity but markedly altered community composition, enriching genera such as *Pantoea* and *Bacillus* that were associated with variation in carbon fractions and aggregate properties.

**Conclusion:**

Overall, HBOF appears to promote a microbially mediated redistribution of SOC and physical protection of newly added labile carbon within macroaggregates, leading to short-term increases in SOC and improved soil structure in red soils. These findings provide a basis for optimizing organic fertilization and carbon management in medium- and low-yield croplands.

## Graphical abstract

**Figure:**
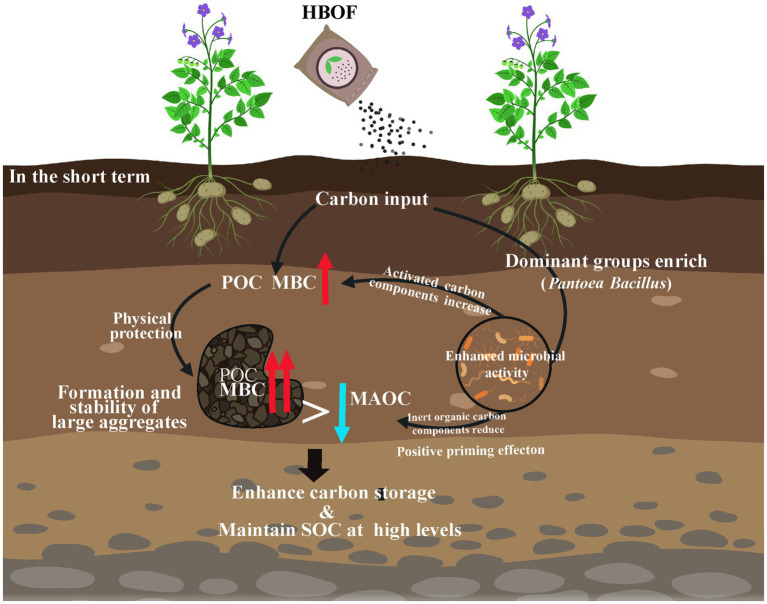

## 1 Introduction

In Yunnan Province, medium- and low-yield fields account for more than 80% of the cultivated red soils, and the average quality grade of farmland is low, indicating substantial potential for productivity improvement. These fields are constrained by limiting factors such as low nutrient content, poor soil structure, and low organic carbon levels, which restrict agricultural production (Bai et al., 2023; Yan et al., 2016; Zhang and Li, 2023). Changes in the soil carbon pool, a crucial indicator for assessing soil organic carbon dynamics, have a direct impact on soil fertility, structure, carbon sequestration capacity, crop yields, and agricultural sustainability, making it a priority for regulation in medium- and low-yield fields (Chen and Shi, 2020; Lal, 2004; Liang et al., 2019; Yan et al., 2016; Yongzan et al., 2015). Soil carbon stocks, which quantitatively represent the soil carbon pool, are regulated by multiple factors, including climatic conditions, land-use type, and anthropogenic activities, and augmenting these stocks is a crucial strategy for achieving soil organic carbon sequestration.

Soil organic carbon (SOC) sequestration is primarily governed by the coupled processes of carbon-fraction transformation and physical protection within soil aggregates (Jiang H. et al., 2025; Yang et al., 2021). Soil aggregates encapsulate organic carbon and provide physical protection that retards its decomposition and thereby promotes SOC sequestration. This protective function differs among aggregate-size classes, with macroaggregates being more favorable for safeguarding labile carbon fractions, whereas microaggregates are more conducive to the long-term stabilization of relatively inert organic carbon (Chandra and Naresh, 2020; Jiang W. et al., 2025). Microorganisms play a central role in these processes. They secrete extracellular enzymes to decompose and transform organic matter, and they assimilate and metabolize substrates to form microbial necromass carbon (MNC), which is stabilized through association with minerals and entombment within aggregates (Schimel et al., 2007; Waldrop and Firestone, 2004). External carbon inputs regulate SOC sequestration by modulating microbial activity and thus the balance between priming and entombing effects. on the one hand, the addition of easily decomposable carbon can stimulate microbial decomposition of pre-existing SOC and increase CO₂ release; on the other hand, enhanced microbial anabolism and higher carbon use efficiency favor the formation of more recalcitrant pools, such as mineral-associated organic carbon (MAOC) and MNC, thereby promoting SOC accumulation (Liang et al., 2017; Nottingham et al., 2009; Zhou et al., 2025). According to the microbial carbon pump theory, organic fertilizers increase microbial activity and extracellular enzyme secretion, facilitating the transformation of labile organic carbon (e.g., particulate organic carbon and microbial biomass carbon) into more stable forms (e.g., MAOC), while simultaneously providing readily decomposable substrates that improve microbial carbon use efficiency and lead to a predominance of carbon carryover and sequestration rather than respiratory loss (Angst et al., 2021; Coban et al., 2022). In addition, enhanced microbial activity stimulates the production of binding agents such as extracellular polymeric substances and polysaccharides, which promote the formation of macroaggregates and increase aggregate stability, thereby strengthening the physical protection of SOC (Bhattacharyya et al., 2021; Liu et al., 2021; Rabbi et al., 2020; Tang and Wang, 2022; Tian et al., 2022). Bio-organic fertilizers can therefore substantially enhance soil carbon sequestration by increasing the abundance, activity and diversity of functional microorganisms, improving carbon use efficiency, facilitating the conversion of labile carbon to recalcitrant carbon, and reinforcing aggregate-mediated physical protection of carbon (Eduah et al., 2025; He et al., 2023; Jin et al., 2022; Niu et al., 2024; Rong et al., 2025). However, conventional bio-organic fertilizers generally have relatively low carbon contents (approximately 30–40%), and the activity of functional microorganisms declines rapidly once the carbon sources in the substrate are depleted, resulting in limited carbon sequestration (Kong and Lu, 2022; Li et al., 2020; Li et al., 2021). Because carbon is the primary energy source for microorganisms, increasing the carbon content of bio-organic fertilizers is expected to alleviate energy and nutrient constraints, enhance microbial carbon use efficiency, and favor SOC sequestration rather than respiratory carbon loss (Sun et al., 2025; Zhou et al., 2025). Therefore, the carbon content of bio-organic fertilizers is likely a key determinant of their effectiveness in promoting soil carbon sequestration.

Consequently, high-carbon bio-organic fertilizers (HBOF), generally characterized by an organic matter content exceeding 40% (Zhang et al., 2021), have garnered increasing attention, particularly in red soil regions. Red soils are typically characterized by high acidity, strong weathering, low organic carbon and nutrient reserves, weak aggregate structure and low biological activity (Baligar et al., 2004; Si et al., 2023). Under such conditions, conventional bio-organic fertilizers, due to their relatively low carbon inputs and primarily long-term carbon accumulation effects, often fail to provide sufficient carbon substrates in the short term to rebuild soil structure or sustain high microbial activity, thereby limiting their positive impacts on soil carbon pools and productivity in medium- and low-yield red-soil croplands (Huang et al., 2010; Sun et al., 2025; Tong et al., 2014). In contrast, HBOF can supply much larger amounts of organic matter in a single application. Research suggests that the application of high-carbon organic fertilizers can concurrently enhance crop growth and development while improving crop quality. Furthermore, these fertilizers have been shown to improve soil physicochemical properties, increase nutrient availability, enhance soil microbial activity, and elevate active carbon content, thereby facilitating the accumulation of organic carbon (Chen et al., 2023; Wei and Li, 2022; Zhang et al., 2018). However, despite these benefits and the well-established role of microorganisms in soil carbon cycling, there is a notable research gap regarding the specific regulatory pathways of HBOF. Unlike conventional organic fertilizers, it remains unclear how HBOF, through its distinct high-carbon input, modulates the interplay between microbial communities and soil aggregates to alter carbon fractions and drive carbon sequestration, particularly in medium- and low-yield fields. Furthermore, the key regulatory factors governing this process remain undefined. This knowledge gap limits our understanding of how HBOF can sustainably increase soil carbon pools while ensuring environmentally friendly yield increases, thereby hindering their further development and adoption.

Based on this context, the study proposes the following two hypotheses. Compared with other organic fertilizers: (1) HBOF further promote organic carbon transformation, enhance the formation and stability of large aggregates, and increase soil carbon storage; (2) HBOF may promote aggregate formation by increasing the relative abundance of functional microorganisms and/or by modulating carbon-degrading microorganisms, thereby influencing carbon fraction transformation and further enhancing soil carbon storage. This study uses potato as the test crop and implements four distinct treatments: chemical fertilizer (CK), organic fertilizer (OF), bio-organic fertilizer (BOF), and high-carbon bio-organic fertilizer (HBOF). By analyzing the dynamics and interrelationships among carbon storage, organic carbon fractions, aggregate characteristics, and microbial community composition, the study aims to: (1) Investigate the impact of HBOF application on soil aggregate characteristics and organic carbon components, elucidating the differences in carbon-enhancing effects between HBOF and other organic fertilizers; and (2) Assess the influence of HBOF on key microbial communities involved in soil aggregate formation. Through correlation analysis and functional prediction, the study further aims to elucidate the potential mechanisms of synergistic carbon enhancement arising from the interaction between carbon inputs from HBOF and microbial activity.

## 2 Materials and methods

### 2.1 Research area and materials

#### 2.1.1 Research area overview

The field experiment was conducted at the Xundian potato cultivation base in Kunming City, Yunnan Province, China (102°55′E, 25°48′N), which has a low-latitude plateau monsoon climate. The site has a mean annual temperature of 17.6 °C, with a maximum temperature of 33.3 °C. The frost-free period averages 218 days per year, and mean annual precipitation is 940 mm. The soil type is upland red soil, with the following fundamental physicochemical properties: organic matter 9.68 g kg^−1^, available nitrogen (AN) 74.5 mg kg^−1^, available phosphorus (AP) 74.5 mg kg^−1^, available potassium (AK) 122.4 mg kg^−1^, and pH 5.6. These characteristics meet the criteria for medium- and low-yield fields in Southwest China.

#### 2.1.2 Experimental materials

The test crop utilized in this study was *Solanum tuberosum* Qing Shu 9. The experimental fertilizers comprised conventional chemical fertilizers, organic fertilizers, bio-organic fertilizers, and high-carbon bio-organic fertilizers. OF and high-carbon organic fertilizer (HOF) were prepared via aerobic composting using cattle manure and straw as raw materials, with initial C/N ratios adjusted to 25:1 for OF and 35:1 for HOF (achieved by increasing the straw proportion). Their basic physicochemical properties are summarized in Table 1. Notably, the organic matter content of HOF reached 54.6%, which is substantially higher than the minimum requirement specified in the Agricultural Industry Standard of China (NY525), thus supporting its classification as a “high-carbon organic fertilizer”. Subsequently, *Bacillus subtilis* was inoculated into the matured OF and HOF substrates, respectively, to initiate a secondary fermentation. After 7 days of continuous incubation, bio-organic fertilizer (BOF) and HBOF (HBOF) were obtained, with viable bacterial counts of 6.14 × 10^8^ cfu g^−1^ and 6.34 × 10^8^ cfu g^−1^, respectively. Other basic properties did not differ significantly from those of the corresponding compost substrates.

**Table 1:** Physicochemical properties of the matured organic fertilizer (OF) and the high-carbon organic fertilizer (HOF).

Fertilizer type	N (%)	P₂O₅ (%)	K₂O (%)	Organic matter (%)	EC (mS cm^−1^)	pH
OF	1.1	1	1.2	37.4	1.39	7.16
HOF	1.8	1.6	1.5	54.6	2.24	7.43

### 2.2 Experimental design

The field experiment included four treatments: chemical fertilizer (CK), organic fertilizer (OF), bio-organic fertilizer (BOF), and high-carbon bio-organic fertilizer (HBOF). The experiment was arranged in a randomized block design with three replicates per treatment, totaling 12 plots. Each plot covered an area of 32 m^2^ (4 m × 8 m). Potatoes were planted at a density of 79,980 plants ha^−1^, with a row spacing of 60 cm and plant spacing of 30 cm. Guard rows were established around the field perimeter, and field management followed standard local farming practices. The CK treatment followed the local conventional fertilization rates (N: 150 kg ha^−1^, P₂O₅: 130 kg ha^−1^, K₂O: 170 kg ha^−1^), using single-nutrient chemical fertilizers (urea, superphosphate, and potassium sulfate). For the organic fertilizer treatments, application rates were adjusted according to a potassium-equivalent substitution strategy rather than a fixed rate. Specifically, OF and HOF were applied at 2,834 kg ha^−1^ and 2,267 kg ha^−1^, respectively, designed to substitute approximately 20% of the total K₂O input with K₂O supplied by organic fertilizer, with the remaining K₂O supplied by potassium sulfate. The nutrient contents (N, P₂O₅, and K₂O) of each organic fertilizer were determined prior to application, and their nutrient contributions were accounted for when calculating the amounts of urea, superphosphate, and potassium sulfate. Mineral fertilizers were supplemented to compensate for any nutrient deficits so that the total inputs of N, P₂O₅, and K₂O were kept comparable across all treatments. Because the objective of this study was to test the effect of increased organic carbon inputs from different organic fertilizers on soil carbon storage, we did not use a carbon-equivalent substitution design, which would homogenize carbon inputs among treatments. Instead, under the potassium-equivalent substitution framework and after adjusting mineral fertilizers to keep total N, P₂O₅, and K₂O inputs comparable across treatments, the organic matter inputs were 1,059 kg ha^−1^ in OF and 1,237 kg ha^−1^ in HOF, providing a clear contrast in organic inputs consistent with our study objective. All fertilizers were applied as basal fertilizer in planting holes prior to sowing.

### 2.3 Soil sampling

Before sowing, soil samples were collected from the 0–20 cm tillage layer using the five-point sampling method. These samples were subsequently homogenized by quartering method to create a composite soil sample for laboratory analysis. Additional soil samples were collected at the maturity stage, 155 days post-sowing. After natural air-drying, the samples were sieved for determination of soil physicochemical properties, including SOC, AN, AP, and AK. Concurrently, rhizosphere soil samples were collected in triplicate for each treatment. These samples were immediately placed in sterile bags and transported to the laboratory on dry ice for microbial analysis. Within each plot, three sampling points were randomly selected to collect undisturbed soil from the 0–20 cm tillage layer. After air-drying, soil aggregate analysis was conducted. Furthermore, bulk density samples were obtained using a 100 cm^3^ cutting ring.

### 2.4 Soil sample analysis parameters

#### 2.4.1 Determination of soil organic carbon fractions, physicochemical properties, and aggregate characteristics

In this study, aggregate-size distribution and stability indices were determined on fractionated soil samples, whereas soil carbon variables and microbial communities were analyzed at the bulk-soil level. Specifically, SOC, CS, carbon fractions and microbial community composition were all measured from bulk soil. Aggregate-size classes and aggregate stability were obtained from aggregate fractionation. Total organic carbon was quantified using the potassium dichromate oxidation method with heating. AN was determined through the alkali-hydrolyzed diffusion technique. AP was evaluated using sodium bicarbonate extraction, followed by the molybdenum-antimony colorimetric method. AK was assessed via ammonium acetate extraction in conjunction with flame photometry. Soil pH was measured with a pH meter after preparing a soil-water mixture at a 1:5 ratio. SOC fractions were separated using the physical fractionation method (Cambardella and Elliott, 1992). Briefly, 10 g of air-dried soil (< 2 mm) was dispersed in 30 mL of 5 g L^−1^ sodium hexametaphosphate solution and shaken on a reciprocating shaker for 18 h to ensure complete dispersion of soil aggregates. The suspension was then passed through a 53 μm sieve and rinsed thoroughly with distilled water until the filtrate became clear. The fraction retained on the sieve (> 53 μm) was defined as particulate organic carbon (POC), while the slurry passing through the sieve (< 53 μm) was collected as MAOC. Both fractions were oven-dried at 60 °C to constant weight, ground, and analyzed for organic carbon content using the potassium dichromate oxidation method with external heating. Soil readily oxidizable organic carbon (ROOC) was measured using potassium permanganate oxidation and ultraviolet spectrophotometry. Microbial biomass carbon (MBC) and dissolved organic carbon (DOC) were prepared using chloroform fumigation and distilled water extraction, respectively, and quantified with a carbon analyzer (TOC-VC, Shimadzu, Kyoto, Japan). Soil aggregates were classified into four distinct particle size fractions (> 2 mm, 1–2 mm, 0.25–1 mm, < 0.25 mm) utilizing the Savinov dry sieving technique in conjunction with Xu’s modified wet sieving approach. For each fraction, the organic carbon content and the organic carbon contribution rate were computed (Xu and Wang, 2014; Yan et al., 2023). In alignment with established research methodologies, the contribution rates of each aggregate size fraction to SOC (Yan et al., 2023) was calculated according to Equation 1:


$$\mathrm{CR}-\mathrm{AO}C_{i}(\%)=\frac{\mathrm{SO}C_{i}\times W_{i}}{\mathrm{SOC}}\times 100\;$$ (1)

CR-AOC_i_, the organic carbon contribution rate of a specific aggregate size class. SOC_i_, the organic carbon content within that aggregate size class. W_i_, the percentage of the total aggregate size class. SOC, the total soil organic carbon content. Soil carbon stocks (CS) (Zheng et al., 2022) was calculated according to Equation 2:


$$\mathrm{MWD}=\sum_{i=1}^{n}{\bar{d}}_{i}\times W_{i}$$ (2)

CS, soil organic carbon stock. SOC, soil organic carbon content. H, soil thickness = 20 cm; BD, bulk density.

Mean weight diameter (MWD), geometric mean diameter (GMD), and percentage of aggregate destruction (PAD) have been identified as critical indicators for assessing soil aggregate stability (Ma et al., 2022). MWD, GMD, and PAD were calculated according to Equations 3,4:


$$\mathrm{GMD}=\exp[\frac{\sum_{i=1}^{n}m_{i}\ln{\bar{d}}_{i}}{\sum_{i}^{n}m_{i}}]$$ (3)

$$\mathrm{PAD}=\frac{W_{d0.25}-W_{w0.25}}{W_{d0.25}}\times 100\%$$ (4)

Soil carbon stocks (CS) (Zheng et al., 2022) was calculated according to Equation 5:


$$\mathrm{CS}(t\;ha^{-1})=S\mathrm{OC}\times H\times \mathrm{BD}\times 0.1\;$$ (5)

MWD, mean weight diameter. GMD, geometric mean diameter. PAD, structural aggregate destruction rate. ${\bar{d}}_{i}$ , the average diameter of water-stable aggregates. W_i_, the percentage of soil aggregate size fraction relative to the total mass. Exp., the exponential function with base e (the natural constant). *m*, the total mass of water-stable aggregates. *m_i_*, the dry weight of aggregate components in a specific size fraction. *W_d0.25_*, the mass of mechanically stable aggregates > 0.25 mm. *W_w0.25_*, the mass of water-stable aggregates > 0.25 mm.

#### 2.4.2 Soil bacterial community analysis

Amplicon sequencing and bioinformatic analyses were performed by Wuhan Metware Biotechnology Co., Ltd. (Wuhan, China). For each treatment, 0.5 g of fresh rhizosphere soil was collected. Total genomic DNA was extracted using a cetyltrimethylammonium bromide (CTAB) protocol according to the standard procedure. DNA quality was checked on 1% agarose gels, and DNA concentration and purity were measured with a NanoDrop One spectrophotometer (Thermo Fisher Scientific, USA). DNA was diluted with sterile water to 1 ng μL^−1^ for PCR. Diluted genomic DNA was used as template. According to the selected sequencing region, barcoded specific primers and Phusion® High-Fidelity PCR Master Mix with GC Buffer (New England Biolabs, USA) were used to ensure amplification efficiency and accuracy. For bacterial community analysis, the V3–V4 region of the 16S rRNA gene was amplified with barcoded primers 341F (5´-CCTAYGGGRBGCASCAG-3′) and 806R (5´-GGACTACNNGGGTATCTAAT-3′). PCR was carried out in 30 μL reactions containing Phusion® Master Mix, 2 μM of each primer and approximately 10 ng template DNA, with an initial denaturation at 98 °C for 1 min; 30 cycles of 98 °C for 10s, 50 °C for 30s and 72 °C for 30s; and a final extension at 72 °C for 5 min. PCR products were checked on 2% agarose gels, purified with magnetic beads, quantified, and pooled in equimolar amounts. Target bands were recovered using a Gel Extraction Kit (Qiagen, Germany). Libraries were prepared with a TruSeq® DNA PCR-Free Sample Preparation Kit (Illumina, USA), quantified by Qubit and qPCR, and sequenced on an Illumina NovaSeq 6,000 platform (Illumina, USA) to generate 250 bp paired-end reads.

### 2.5 Data analysis

Statistical analyses were performed using SPSS Statistics 27, with the level of significance established at *p* ≤ 0.05. Graphical representations were created using Origin version 25b software. To identify the key soil carbon fractions associated with carbon storage (CS), a random forest regression model was implemented in R (version 4.5.2) using the packages randomForest and rfPermute. CS was used as the response variable and soil carbon fractions (e.g., ROOC, POC, MAOC, DOC) were included as predictors. The random forest model was fitted with 1,000 trees (ntree = 1,000), using the default settings for mtry. Variable importance was evaluated based on the percentage increase in mean squared error (%IncMSE), and permutation tests were conducted using the rfPermute function (ntree = 1,000, nrep = 299, num.cores = 2) to assess the statistical significance of variable importance. Predictors with significant %IncMSE (*p* < 0.05) were considered key carbon fractions influencing CS. The importance values and associated *p*-values were extracted and visualised using ggplot2, with significance levels indicated by asterisks (*p < 0.05, **p < 0.01, ***p < 0.001). Subsequently, multiple linear regression was used to evaluate the relative importance of aggregate characteristics in explaining variation in soil carbon fractions. In the initial models, soil aggregate indices were included as explanatory variables and soil carbon fractions were used as response variables. Multicollinearity among aggregate predictors was assessed using tolerance and variance inflation factors (VIF). When VIF values exceeded 10 for certain variables, indicating severe multicollinearity, the set of aggregate variables was reduced. To analyse the relationships between aggregate characteristics and soil carbon fractions while controlling for multicollinearity, VIF-based screening was applied to aggregate-related variables. Because the proportion of macroaggregates (> 2 mm) showed strong collinearity with MWD and GMD, these three variables were not included simultaneously. Instead, PAD was selected as an indicator of aggregate stability, and the proportions of aggregates < 0.25 mm and 0.25–1 mm were used to represent aggregate-size distribution. For this refined set of aggregate predictors, VIF values ranged from 1.1 to 3.7, indicating acceptable levels of multicollinearity. Based on the key carbon fractions identified by the random forest analysis and the screened aggregate indices, redundancy analysis (RDA) was performed using Canoco 5 to examine the relationships among aggregate characteristics, soil carbon fractions, and CS, and to evaluate the associations between these variables and soil microbial genera. Microbial community *α*-diversity was assessed using the Chao, Shannon, and Simpson indices to evaluate community richness and diversity. *β*-diversity was analyzed based on Euclidean distance matrices to examine the variation in microbial community composition among different treatments. Principal coordinates analysis (PCoA) was subsequently performed on the Euclidean distance matrices using the Metware Cloud (a free online platform for data analysis, https://cloud.metware.cn), and the first two principal coordinates were used to visualize differences in community structure among treatments. Functional abundance predictions based on the microbial abundance table were carried out using FAPROTAX version 1.2.6, resulting in relative abundances for 71 functions, which were subsequently categorized into 12 metabolic pathways. Origin version 25b was also utilized for correlation analysis among the metabolic pathways, as well as for clustering and visualization of functional relative abundances across the four treatment groups. The graphical abstract was created using BioRender.^1^

## 3 Results

### 3.1 Soil aggregate characteristics

**Figure 1:**
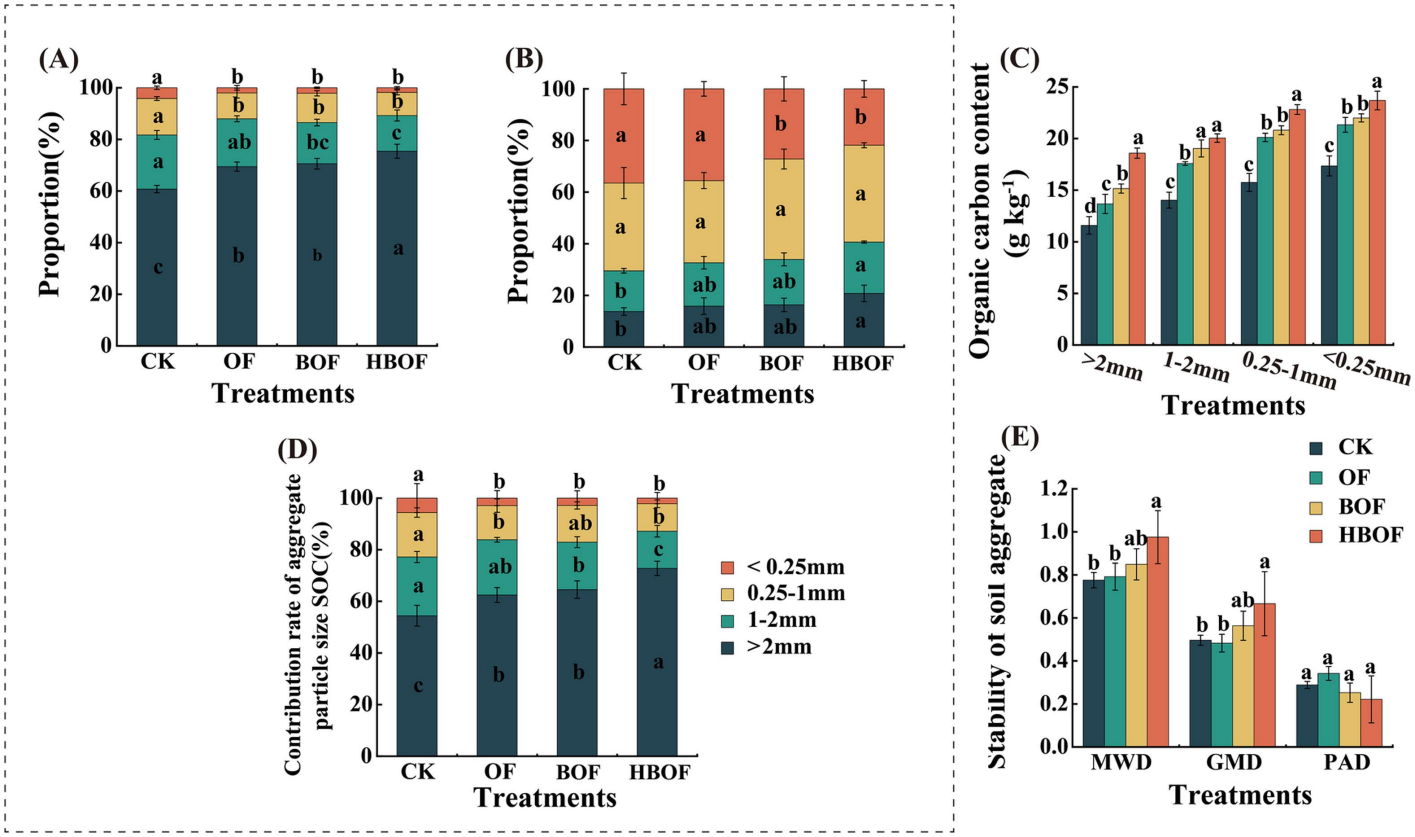
Effects of applying different organic fertilizers on soil aggregates **(A)** particle size composition of mechanical aggregates; **(B)** particle size composition of water-stable aggregates; **(C)** organic carbon in soil aggregates of various particle sizes; **(D)** contribution rate of organic carbon of each particle size in soil aggregates; **(E)** aggregate stability. MWD, the average weight diameter. GMD, the geometric weight diameter. PAD, the failure rate of the structure. Different lowercase letters above bars indicate significant differences among treatments (*p* < 0.05, Tukey’s HSD test). Error bars represent standard deviation (*n* = 3 biological replicates).

Different organic fertilizer treatments significantly promoted the formation of large soil aggregates and enhanced their stability. Additionally, these treatments increased the organic carbon content across all particle sizes within aggregates and their contribution rate to total organic carbon (Figure 1). Specifically, the HBOF treatment significantly increased the proportion of mechanically stable aggregates with aggregates > 2 mm by 6.87 and 8.58% compared to the BOF and OF treatments, respectively, although it did not significantly affect the content of water-stable aggregates (Figures 1A,B, *p* < 0.05). Across all treatments, the organic carbon content within soil aggregates increased as particle size decreased. The HBOF treatment exhibited the highest organic carbon content across all particle sizes, followed by the BOF and OF treatments, with the CK treatment had the lowest values (Figure 1C). The relative contribution of organic carbon within aggregates larger than 2 mm to the total SOC was found to increase with higher levels of exogenous carbon input. The most substantial relative contribution, reaching 72.79%, was observed in large aggregates under the HBOF treatment. In contrast, the relative contributions from other particle size fractions diminished as carbon input increased (Figure 1D, *p* < 0.05). Furthermore, both MWD and GMD under the HBOF increased significantly by 24.53 and 36.15%, respectively, compared with the CK and OF treatments (Figure 1E, *p* < 0.05). However, the different fertilization treatments did not have a significant impact on the PAD values of soil aggregates (Figure 1E).

### 3.2 Soil organic carbon fractions, carbon stock, and key regulatory factors

**Figure 2:**
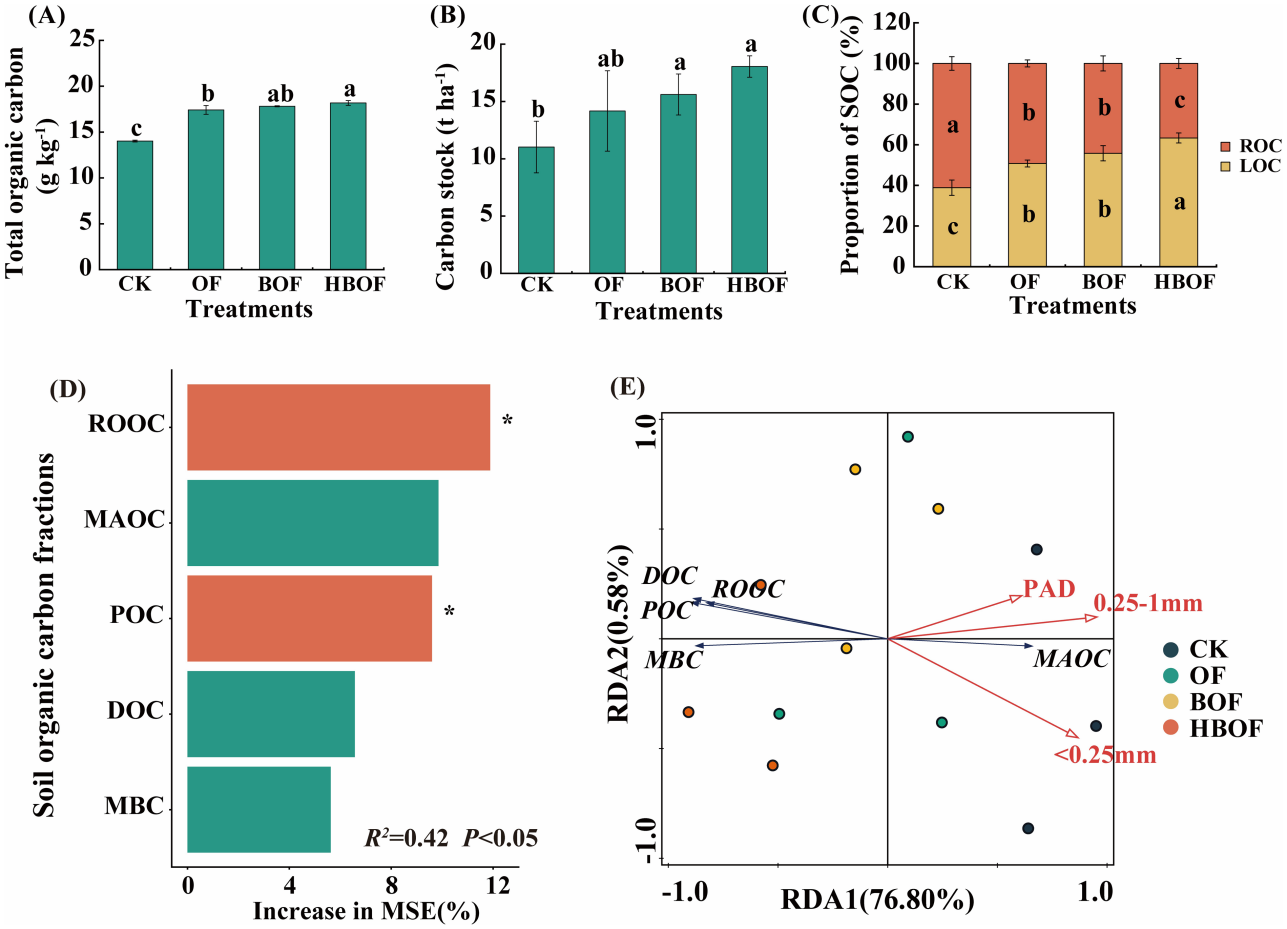
Effects of fertilization regimes on soil carbon fractions and key drivers of carbon sequestration. **(A,B)** Effects of different treatments on total organic carbon and carbon storage; **(C)** Contribution of organic carbon fractions to TOC under different treatments; **(D)** Random forest model identifies the key carbon fractions driving carbon stock accumulation (*p < 0.05); **(E)** RDA reveals the associations between aggregate characteristics and carbon fractions. TOC, Total Organic Carbon; CS, Carbon Stock; LOC, Labile Organic Carbon; ROC, Recalcitrant Organic Carbon; POC, Particulate Organic Carbon; MAOC, Mineral-Associated Organic Carbon; MBC, Microbial Biomass Carbon; DOC, Dissolved Organic Carbon; ROOC, Readily Oxidizable Organic Carbon. Different lowercase letters above bars indicate significant differences among treatments (*p* < 0.05, Tukey’s HSD test). Error bars represent standard deviation (*n* = 3 biological replicates).

In comparison to the CK treatment, the application of various organic fertilizers significantly increased active soil carbon, particularly POC, and reduced inert organic carbon, such as MAOC (Supplementary Figure S1). Furthermore, this application significantly increased the contents of organic carbon with varying chemical activities, including DOC, ROOC, and MBC. Compared with the other treatments, TOC under HBOF increased by 2.02–29.62%, and CS significantly increased by 63.71% relative to CK, although CS under HBOF did not differ significantly from that under the OF and BOF treatments (Figures 2A,B, *p* < 0.05). The application of HBOF significantly enhanced the proportion of active organic carbon relative to TOC, while reducing the proportion of inert organic carbon (Figure 2C). Random forest analysis indicated that among soil carbon fractions, ROOC and POC were the key factors driving CS accumulation (contributions of 11.88 and 9.60%, respectively; *R^2^* = 0.42, *p* < 0.05; Figure 2D). RDA further showed that PAD and the proportions of aggregates < 0.25 mm and 0.25–1 mm were significantly negatively correlated with all labile organic carbon fractions, with 0.25–1 mm aggregates identified as the dominant factor influencing SOC fractions (contribution of 90.2%, *p* = 0.002; Figure 2E). These results indicate that small aggregates make a major contribution to the variation in soil carbon fractions. Because the proportion of aggregates > 2 mm was strongly collinear with other aggregate characteristics, this variable was excluded from the RDA to avoid multicollinearity. Nevertheless, given its close association with MWD and GMD and the well-documented role of macroaggregates in protecting and stabilizing SOC, the formation of large aggregates is still likely to play a crucial role in regulating soil carbon fractions. Consequently, although MAOC content under HBOF decreased relative to CK, POC increased markedly, and CS under HBOF remained significantly higher than under the other treatments and did not differ significantly from that under OF and BOF (Figures 2A,B; Supplementary Figure S1, *p* < 0.05).

### 3.3 Alterations in soil bacterial community characteristics

**Figure 3:**
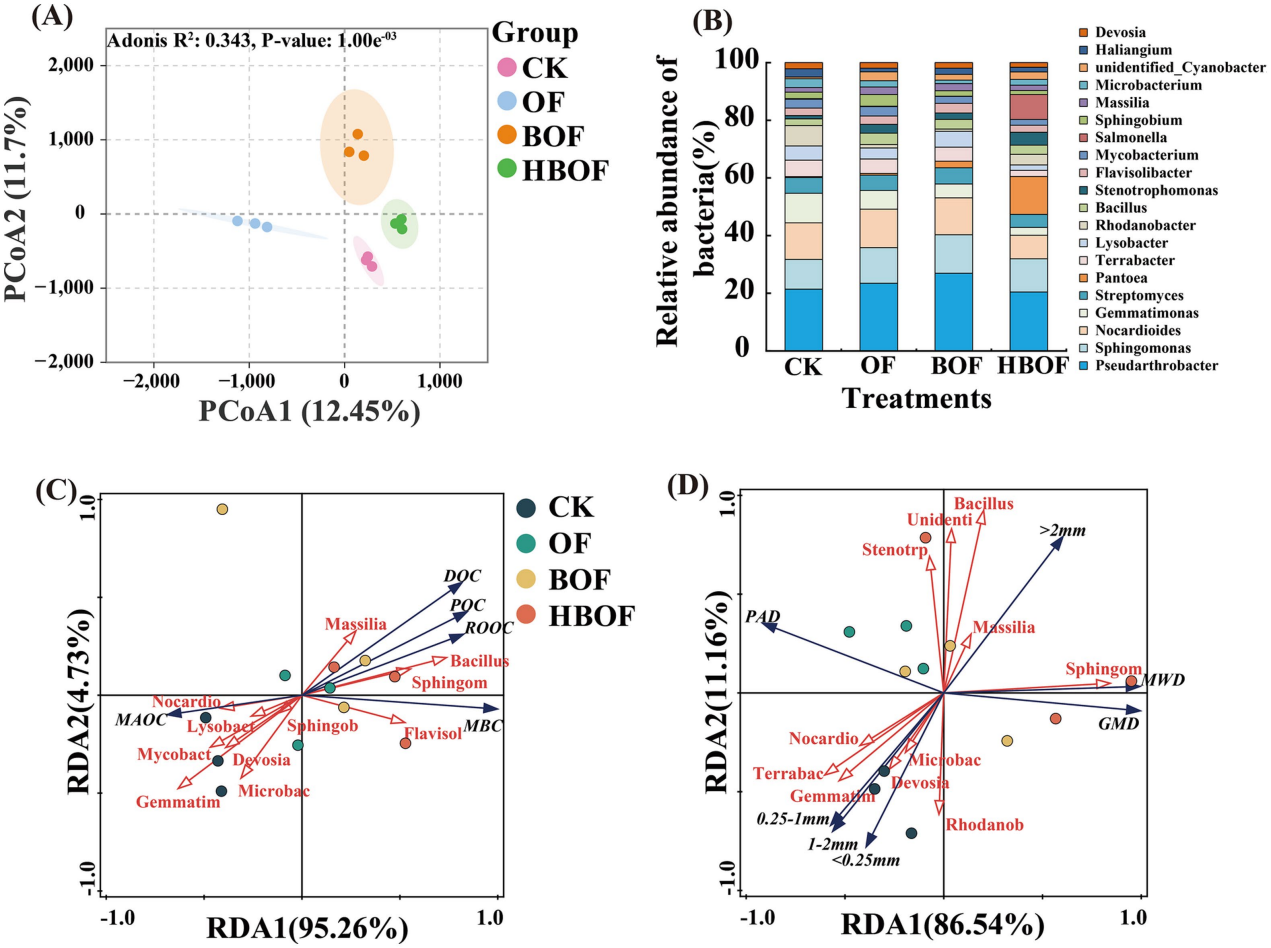
Effects of fertilization treatments on soil bacterial communities. **(A)** PCA reveals significant differences in bacterial communities among treatments. **(B)** Variations in relative abundance of bacterial taxa at genus level. **(C,D)** RDA demonstrates: relationships between top 20 bacterial taxa (relative abundance) and carbon fractions/aggregate characteristics.

Compared with other treatments, HBOF treatment significantly reduced the Chao index, Shannon index, and Simpson index (Supplementary Table S1, *p* < 0.05), indicating that HBOF application markedly decreased soil bacterial community diversity. PCoA demonstrated that different treatments significantly influenced the composition of the bacterial community, with PCoA1 and PCoA2 explaining 12.45 and 11.70% of the variance, respectively, cumulatively accounting for 24.15% (Figure 3A, *P_adonis_* = 0.001). At the phylum level, the HBOF treatment significantly increased the relative abundances of *Firmicutes* and *Proteobacteria*, while substantially decreasing that of *Gemmatimonadota* (Supplementary Figure S2A, *p* < 0.05). At the genus level, HBOF application markedly enriched the genera *Bacillus* and *Pantoea* in comparison to other treatments (Figure 3B, *p* < 0.05). RDA revealed that at the phylum level, the dominant groups main biological factor affecting carbon fractions variation were *Firmicutes* and *Bacteroidetes*, which explained 58.1% (*p* = 0.004) and 23.2% (*p* = 0.008) of the variation, respectively (Supplementary Figure S2B). At the genus level, *Bacillus* emerged as the principal biological factor affecting carbon fraction, accounting for 52% of the variation (*p* = 0.008, Figure 3C). Furthermore, *Sphingomonas* and *Bacillus* were key biological factors influencing the formation of aggregates larger than 2 mm, (contributing 61.5%, *p* = 0.008 and 11.8%, *p* = 0.002, respectively; Figure 3D).

### 3.4 Soil microbial functions and correlations with soil physicochemical properties

**Figure 4:**
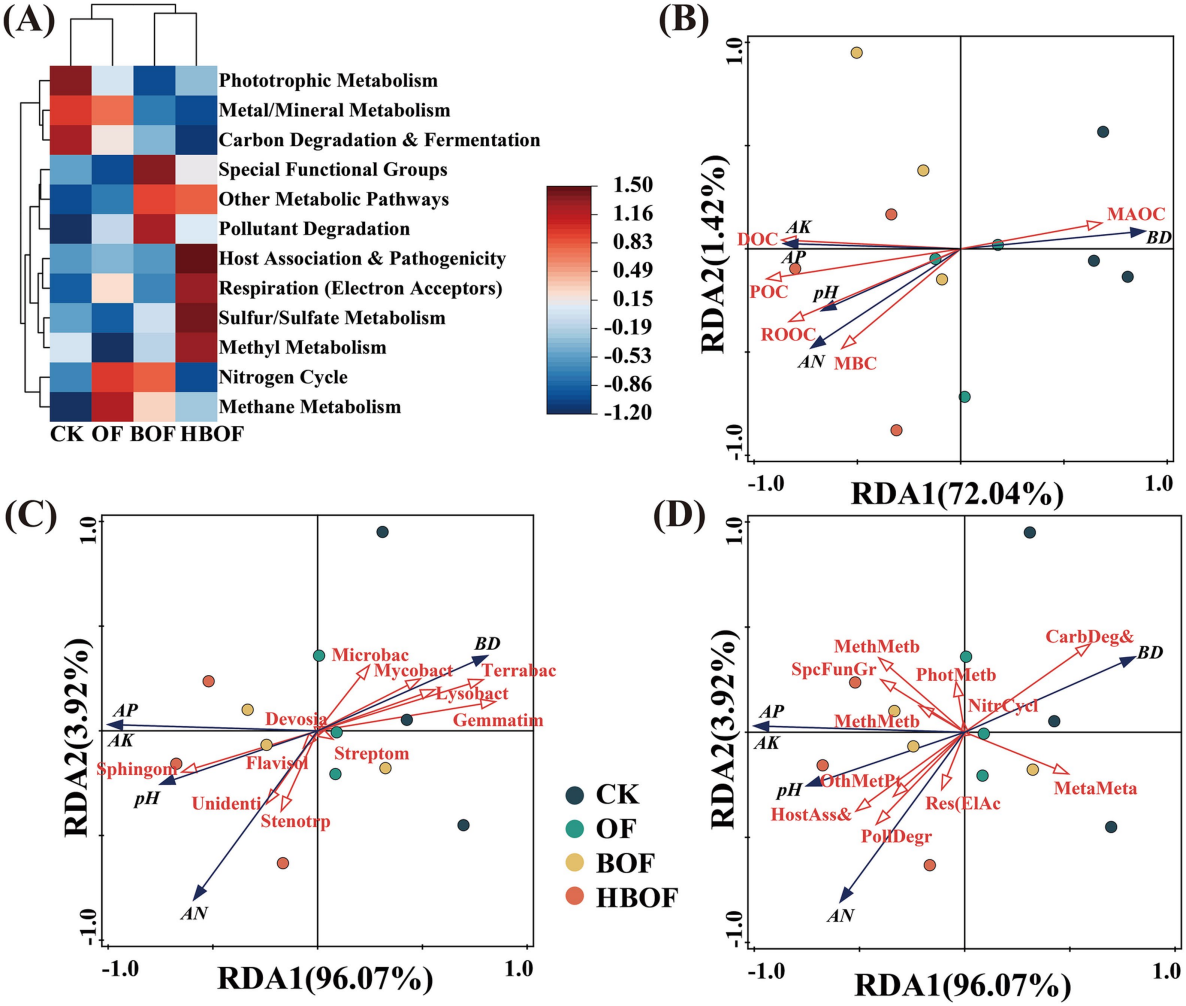
Functional divergence among treatments and key environmental drivers. **(A)** Functional prediction based on FAPROTAX: analysis of functional abundance among different treatments. **(B–D)** RDA reveals the relationships of carbon fractions, the top 20 bacterial genera (relative abundance), and metabolic functions with the soil environment. pH, soil acidity/alkalinity; AN, available nitrogen; AP, available phosphorus; AK, available potassium; BD, bulk density.

Functional predictions using FAPROTAX indicated that HBOF significantly increased the relative abundances of bacterial functional groups associated with respiration, potential pathogenicity, sulfur/sulfate metabolism, and methyl metabolism compared with the other treatments. In contrast, the relative abundances of functions related to metal/mineral metabolism, carbon degradation, and nitrogen cycling showed the opposite trend (Figure 4A, *p* < 0.05). RDA revealed that variations in soil physicochemical properties across different fertilization treatments was mainly explained by the abiotic factor POC (contribution 86.4%, *p* = 0.002; Figure 4B) and by two biotic factors: *Gemmatimonas* (contribution 69.1%, *p* = 0.002; Figure 4C) and the relative abundance of carbon degradation functions (contribution 34.8%, *p* = 0.026; Figure 4D).

## 4 Discussion

### 4.1 Effects of different fertilization treatments on soil aggregate characteristics

Soil aggregates are pivotal in the sequestration and conservation of organic carbon, serving as the primary reservoirs for SOC (Lehmann et al., 2020). Within the context of agricultural soil management, the application of organic fertilizers has been shown to influence the distribution of particle sizes and the stability of soil aggregates (Yilmaz and Sönmez, 2017). The findings of this study corroborate previous researches (Feng et al., 2025; Saccà et al., 2026; Yilmaz and Sönmez, 2017), demonstrating that application of different organic fertilizers promotes the formation of large soil aggregates and significantly enhances aggregate stability (Figure 1). The input of exogenous organic carbon into the soil via organic fertilizers facilitates the binding of organic matter with minerals through synergistic physical, biological, and chemical processes, leading to the formation of microaggregates (Lin et al., 2025; Wu et al., 2024). These microaggregates subsequently coalesce into larger aggregates under the cementing influence of binding agents, such as microbial secretions and organic materials (Lehmann et al., 2017; Six et al., 2000; Wang et al., 2024). This process ultimately augments the structural stability of the soil (Bhattacharyya et al., 2021; Liu et al., 2021; Rabbi et al., 2020). In our study, HBOF further increased the proportion of macroaggregates compared with OF and BOF and enhanced the organic carbon content within aggregates across all particle-size classes, leading to a higher contribution of large aggregates to total SOC (Figure 1). These results indicate that, under the conditions of this experiment, supplementing bio-organic fertilizer with additional organic carbon input can further promote the formation of large soil aggregates and concentrate SOC within them, which is expected to strengthen the physical protection of organic carbon.

### 4.2 Effects of different fertilization treatments on soil organic carbon fractions and carbon storage

Different SOC fractions contribute to carbon cycling over different time scales. MBC, DOC and ROOC represent highly active pools with rapid turnover and act as immediate substrates and drivers of microbial processes (Jiang et al., 2006; Xu et al., 2011). POC, derived mainly from plant and microbial residues, is less active but still relatively labile and serves as an intermediate pool in SOC turnover as well as an important component for aggregate formation (Cheng et al., 2024; Lavallee et al., 2020; Si et al., 2024). In contrast, MAOC is strongly bound to minerals and is generally more resistant to decomposition, thus constituting a key form of long-term carbon sequestration (Haddix et al., 2016; Liang et al., 2019). In this study, all organic fertilizer treatments increased active carbon fractions and CS compared with CK (Figures 2A–C; Supplementary Figure S1), indicating that organic inputs stimulated microbial activity and carbon cycling. The random forest analysis identified ROOC and POC as the strongest predictors of CS accumulation (Figure 2D), highlighting the importance of short-lived, labile pools in explaining treatment differences in CS at the time scale of this experiment. Consistently, RDA showed that PAD and the proportions of aggregates < 0.25 mm and 0.25–1 mm were significantly negatively correlated with labile carbon fractions, and that 0.25–1 mm aggregates accounted for most of the explained variation in SOC fractions (Figure 2E). Together, these results suggest that small aggregates made a major contribution to the observed variation in SOC fractions, likely because they provide extensive mineral surfaces and microhabitats that regulate microbial processing and the exchange between particulate and mineral-associated pools. It should be noted that the proportion of aggregates > 2 mm was excluded from the RDA due to strong collinearity with other aggregate metrics. Therefore, the RDA results should not be interpreted as evidence that macroaggregates are unimportant. Macroaggregates remain closely linked to MWD and GMD and are widely recognized as key structural units that can physically occlude particulate organic matter and influence SOC stabilization pathways. In our data, HBOF increased POC but decreased MAOC compared with CK, leading to a higher POC/MAOC ratio (Figure 2C; Supplementary Figure S1). This pattern points to a short-term trade-off between the amount and stability of sequestered carbon. Although total soil CS under HBOF was significantly higher than under CK and comparable to that under OF and BOF, the accompanying decline in MAOC indicates that part of the native, mineral-associated carbon pool was mobilized. This is consistent with a positive priming effect induced by high-carbon inputs (Mi et al., 2025): the massive input of labile organic carbon likely stimulated microbial activity and prompted microorganisms to decompose native, mineral-protected organic matter to acquire energy and limiting nutrients (Angst et al., 2021; Coban et al., 2022; Nottingham et al., 2009; Qu et al., 2019). Compared with BOF, the higher carbon input in HBOF may have intensified this ‘microbial mining’ of the stable pool. Meanwhile, the rapid accumulation of POC under HBOF suggests that macroaggregates provided effective physical protection for newly added particulate organic matter, whereas transformation from POC to MAOC likely requires a longer turnover time. At the relatively short time scale of this study, the formation of new MAOC was clearly not sufficient to offset the losses caused by the priming effect. Therefore, in the short term, HBOF significantly improved soil fertility and structure by increasing POC and other active carbon fractions. Its main advantage does not lie in providing a further increase in total carbon relative to other organic fertilizers, but rather in reshaping the distribution of SOC among different fractions and structural domains—allocating more carbon into forms that are favorable for aggregate stability and soil fertility, such as POC and other active pools within macroaggregates. However, the long-term stability of sequestered carbon still depends on the subsequent conversion of POC into MAOC. Future long-term field experiments are needed to determine whether the initial SOC gains under HBOF can be maintained once losses of old MAOC are fully taken into account.

### 4.3 Effects of different fertilization treatments on soil bacterial community structure and regulation of soil carbon cycling

Bacteria, as integral components of soil microbial communities, play a crucial role in the cycling of SOC and nutrients. Fertilization practices have been shown to modify the structure of soil bacterial communities (Ibrahim et al., 2021; Kong et al., 2024; Li et al., 2024). The results from this study demonstrate that the application of HBOF resulted in a decrease in soil bacterial community diversity (Supplementary Table S1). This observation suggests that application of HBOF promotes the proliferation of specific key populations, which competed with the native soil bacterial community, thereby reducing diversity (Pan et al., 2025; Pu et al., 2022). PCoA indicated that the different fertilization treatments significantly altered bacterial community composition (Figure 3A). At the phylum level, the HBOF treatment, which included inoculation with *Bacillus subtilis* (*Firmicutes*), increased the relative abundance of *Firmicutes*, suggesting that HBOF created a more favorable environment for functional microorganisms. HBOF also increased the relative abundance of *Proteobacteria* (Supplementary Figure S2A); these phyla have been reported to participate in carbon cycling and aggregate-related processes (Abán et al., 2025; Peng et al., 2025). At the genus level, HBOF enriched *Bacillus* and *Pantoea*, and RDA identified *Sphingomonas* and *Bacillus* as key genera closely associated with variation in carbon fractions and aggregate characteristics (Figures 3C,D). Previous studies have shown that genera such as *Bacillus* and *Pantoea* can produce extracellular metabolites that enhance aggregate cohesion and thereby improve soil structural stability (Hosseinpour et al., 2021; Yang X. et al., 2025). *Sphingomonas* is an important degrader of organic compounds that can influence DOC dynamics and is closely linked to denitrification processes (Liu et al., 2022; Yang et al., 2024). *Gemmatimonas* has been associated with organic matter mineralization in several studies and is often considered unfavorable for carbon sequestration (Yang K. et al., 2025). In our study, HBOF decreased the abundance of *Gemmatimonas* while increasing the relative abundances of *Pantoea*, *Sphingomonas* and *Bacillus*; these changes may jointly contribute to enhanced aggregate formation and enlarged active carbon pools under HBOF. However, we did not directly measure specific microbial products such as extracellular polymeric substances (EPS). Therefore, the microbial mechanisms inferred from community compositional shifts remain preliminary and require confirmation by direct measurements in future work.

Functional prediction based on FAPROTAX showed that, compared with the chemical fertilizer treatment, all three organic fertilizer treatments (HBOF, BOF, and OF) had lower relative abundances of predicted functions related to carbon degradation and nitrogen cycling, with HBOF exhibiting the lowest values (Figure 4A). On the surface, this pattern might suggest that certain carbon-degradation pathways were weakened under organic fertilization. However, FAPROTAX infers functions from the taxonomic identity of cultured reference strains and cannot fully capture the metabolic diversity of uncultured soil microorganisms or their local adaptation processes (Louca et al., 2016). In this study, the FAPROTAX-inferred decline in “carbon-degradation functions” was inconsistent with the observed decrease in MAOC and the increases in MBC and POC, which more strongly indicate enhanced overall microbial activity and the presence of a positive priming effect under HBOF. This inconsistency demonstrates that taxonomy-based functional annotation reflects only part of the heterotrophic processes in soil and cannot, on its own, be taken as evidence that overall carbon degradation is suppressed.

While this study investigated the effects of HBOF on soil carbon storage and its influence on the structure of soil microbial communities, certain limitations persist. Firstly, as the research was conducted as a short-term experiment, it poses challenges in assessing the long-term impacts of HBOF application on SOC fractions and microbial communities. Consequently, long-term field trials are essential to explore these differential effects comprehensively. Secondly, the study inferred potential associations between microbial communities and carbon storage through correlation and RDA but did not quantitatively elucidate specific pathways of carbon-microbe interactions. Future research should employ a diverse array of methodologies, such as microbial co-occurrence networks and structural equation modeling, to uncover the synergistic mechanisms that connect “carbon input-microbial function-organic carbon stabilization.

Consistent with the two a priori hypotheses proposed in the Introduction, our results provide different levels of support. The first hypothesis—that HBOF, relative to other organic fertilizers, would promote organic carbon transformation, enhance the formation and stability of large aggregates, and increase soil carbon storage—was broadly supported. HBOF significantly increased the proportion of macroaggregates, improved aggregate stability indices, and enhanced soil CS compared with CK, while maintaining carbon stocks at levels comparable to the other organic fertilizer treatments. By contrast, the second hypothesis—that HBOF would regulate carbon-fraction transformation and carbon sequestration via shifts in functionally relevant microbial groups, including carbon-degrading microorganisms—was only partially supported by our data. HBOF clearly altered bacterial community composition and was associated with changes in POC and MAOC, which is consistent with a microbially mediated pathway of carbon transformation. However, the FAPROTAX-inferred decline in carbon-degradation functions did not align with the observed decrease in MAOC and increase in MBC and POC, which together indicate a positive priming effect and accelerated decomposition of native stable carbon. This inconsistency suggests that taxonomy-based functional annotation only captures part of the heterotrophic processes in soil and should be interpreted with caution. Overall, our findings support a carbon-sequestration advantage of HBOF in the short term through increased POC accumulation and improved aggregate structure, while also highlighting the need for direct functional measurements to clarify the underlying microbial mechanisms.

## 5 Conclusion

In this short-term field experiment on red soil, all organic fertilizer treatments significantly increased the proportion of macroaggregates, aggregate stability, and various active carbon fractions compared with the unfertilized control. In particular, the HBOF treatment increased the proportion of macroaggregates and, relative to CK, significantly enhanced CS, while maintaining SOC at similarly high levels to those under the OF and BOF treatments. At the same time, HBOF increased POC and MBC and decreased MAOC, indicating a shift of SOC from more inert to more active fractions. These results suggest that, under high-carbon input, HBOF promotes a microbially mediated redistribution of SOC: on the one hand, a positive priming effect leads to the decomposition of part of the pre-existing stable carbon; on the other hand, newly added carbon accumulates in relatively labile forms that receive a certain degree of physical protection within macroaggregates. In the short term, this process leads to a net increase in SOC and its maintenance at relatively high levels, while improving soil structure and fertility. However, the long-term stability of this additional carbon depends on the subsequent transformation of POC into MAOC and on the balance between priming-induced carbon losses and continued carbon inputs. In addition, HBOF markedly reshaped the soil bacterial community, enriching genera such as *Bacillus*, *Pantoea* and *Sphingomonas*, which are associated with carbon fractions and aggregate characteristics, and reducing the abundance of *Gemmatimonas*. Nevertheless, functional predictions based on FAPROTAX and inferences concerning microbial products such as extracellular metabolites remain preliminary and require confirmation by direct functional measurements. Overall, HBOF appears to be a promising management option for rapidly increasing SOC content and improving soil structure in red soils in the short term, but its long-term carbon-sequestration potential still needs to be evaluated through long-term field experiments and more refined functional analyses.

## Data Availability

The raw sequence data reported in this paper have been deposited in the Genome Sequence Archive (Genomics, Proteomics & Bioinformatics 2025) in National Genomics Data Center (Nucleic Acids Res 2025), China National Center for Bioinformation / Beijing Institute of Genomics, Chinese Academy of Sciences (GSA: CRA039676) that are publicly accessible at https://ngdc.cncb.ac.cn/gsa.

## Footnotes

1
https://BioRender.com
