# Supplementary material for: High-carbon bio-organic fertilizer reshapes soil carbon fractions and aggregate structure while maintaining high carbon stocks in red soil
Source: Front Microbiol. 2026 Mar 20;17:1775969. doi: 10.3389/fmicb.2026.1775969 (PMC13047207; doi:10.3389/fmicb.2026.1775969)

Supplementary Material

1. **Supplementary Table 1.** Soil bacterial community alpha diversity indices under different treatments

Treatment	Chao index	Shannon index	Simpson index
CK	4620.15±176.64 a	6.98±0.11 a	0.9960±0.0010a
OF	4454.63±267.52 ab	6.91±0.04 a	0.9950±0.0010a
BOF	4090.23±423.68 ab	6.96±0.14 a	0.9950±0.0017a
HBOF	4028.81±222.68 b	6.57±0.11 b	0.9903±0.0012b

Note: Different lowercase letters indicate significant differences ($P < 0.05$) in soil bacterial alpha diversity among treatments.

2. **Supplementary Table 2.** Phylum-level Relative Abundance: Mean, Standard Deviation, and Statistical Significance.

	Verrucomicrobiota	Myxococcota	Firmicutes
CK	0.0124±0.0057b	0.0124±0.0057b	0.0192±0.0006c
OF	0.013±0.0019ab	0.013±0.0019ab	0.0246±0.0018b
BOF	0.0236±0.0077a	0.0236±0.0077a	0.0307±0.0015a
HBOF	0.0112±0.0059b	0.0112±0.0059b	0.031±0.0046a

	Gemmatimonadota	Acidobacteriota	Actinobacteriota
CK	0.0538±0.0076a	0.0373±0.0042b	0.0459±0.007a
OF	0.0346±0.0071b	0.0283±0.004b	0.038±0.0021ab
BOF	0.0259±0.0041bc	0.0464±0.0066a	0.0352±0.0037b
HBOF	0.0199±0.0087c	0.0342±0.0035b	0.0322±0.006b

	Bacteroidota	Others	unidentified_Bacteria
CK	0.0547±0.01a	0.0513±0.0071b	0.089±0.0228c
OF	0.0655±0.0076a	0.0726±0.0072ab	0.1317±0.0049a
BOF	0.0561±0.0143a	0.0799±0.0191a	0.1214±0.0059ab
HBOF	0.0653±0.0072a	0.0533±0.0097b	0.1015±0.0175bc

	Actinobacteria	Proteobacteria
CK	0.2721±0.0342a	0.3418±0.0225ab
OF	0.2657±0.0194a	0.3115±0.0107b
BOF	0.2534±0.0283a	0.3039±0.0436b
HBOF	0.2412±0.0219a	0.3933±0.0438a

3. **Supplementary Table 3.** Genus-level Relative Abundance: Standard Deviation and Statistical Significance.

	Pseudarthrobacter	Sphingomonas	Nocardioides
CK	0.0613±0.0176a	0.0295±0.0027a	0.0363±0.0026a
OF	0.0687±0.0131a	0.0362±0.0051a	0.0391±0.0054a
BOF	0.0747±0.0126a	0.037±0.0058a	0.0355±0.0007a
HBOF	0.0718±0.0135a	0.0405±0.0097a	0.0286±0.0038b

	Gemmatimonas	Streptomyces	Pantoea
CK	0.0294±0.0057a	0.0156±0.0004a	0.0009±0.0005b
OF	0.019±0.0047b	0.0155±0.002a	0.0018±0.0013b
BOF	0.0133±0.0033b	0.0156±0.0019a	0.0064±0.0004b
HBOF	0.0096±0.0073b	0.0158±0.0004a	0.0461±0.0145a

	Terrabacter	Lysobacter	Rhodanobacter
CK	0.0164±0.0013a	0.014±0.0012a	0.0203±0.0022a
OF	0.0146±0.0025a	0.0112±0.0012ab	0.0035±0.0011c
BOF	0.0135±0.0015a	0.0152±0.0028a	0.0022±0.0001c
HBOF	0.0075±0.0026b	0.0066±0.0059b	0.0129±0.003b

	Bacillus	Stenotrophomonas	Flavisolibacter
CK	0.0066±0.0007c	0.0032±0.0019a	0.0074±0.0007a
OF	0.0115±0.0003a	0.0089±0.0029a	0.0086±0.0028a
BOF	0.0092±0.0009b	0.0061±0.0041a	0.0094±0.0034a
HBOF	0.0112±0.0017ab	0.0157±0.0208a	0.0085±0.0028a

	Mycobacterium	Salmonella	Sphingobium
CK	0.0089±0.0003a	0.0007±0.0003b	0.0062±0.0007b
OF	0.0096±0.0009a	0.0003±0.0004b	0.0118±0.0027a
BOF	0.0067±0.0008b	0.0002±0.0001b	0.0051±0.0012b
HBOF	0.0072±0.0004b	0.0302±0.0119a	0.0049±0.0006b

	Massilia	Microbacterium	unidentified_Cyanobacteria
CK	0.0047±0.0008b	0.0087±0.0012a	0.0016±0.0011a
OF	0.0077±0.0008a	0.0064±0.002a	0.009±0.0018a
BOF	0.0069±0.0008ab	0.0033±0.0005b	0.0056±0.0008a
HBOF	0.0065±0.002ab	0.0071±0.0003a	0.0091±0.0084a

	Haliangium	Devosia
CK	0.0082±0.0017a	0.0061±0.0003a
OF	0.0037±0.0005a	0.0056±0.0006c
BOF	0.0059±0.0004a	0.0054±0.0005b
HBOF	0.0056±0.001a	0.0057±0.0011bc

4. **Supplementary Figure 1.** Effects of fertilization treatments on soil carbon fractions (A-C) Effects of different treatments on labile carbon pool; (D) Effects of different treatments on intermediate carbon pool; (E) Effects of different treatments on recalcitrant carbon pool.

Note: POC, Particulate Organic Carbon. MAOC, Mineral-Associated Organic Carbon. MBC, Microbial Biomass Carbon. DOC, Dissolved Organic Carbon. ROOC, Readily Oxidizable Organic Carbon.

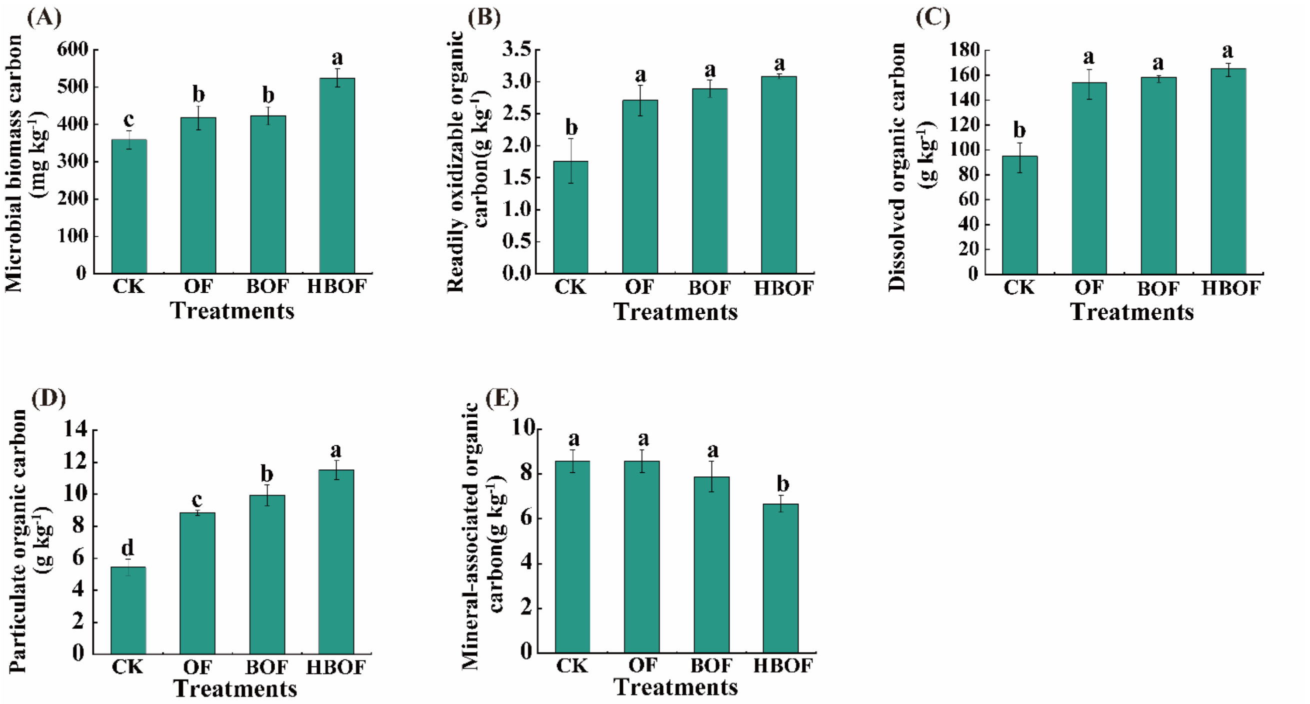

5. **Supplementary Figure 2** Differences in phylum-level relative abundance among treatments (A) and their correlations with carbon fractions (B).

Note: POC, Particulate Organic Carbon. MAOC, Mineral-Associated Organic Carbon. MBC, Microbial Biomass Carbon. DOC, Dissolved Organic Carbon. ROOC, Readily Oxidizable Organic Carbon.

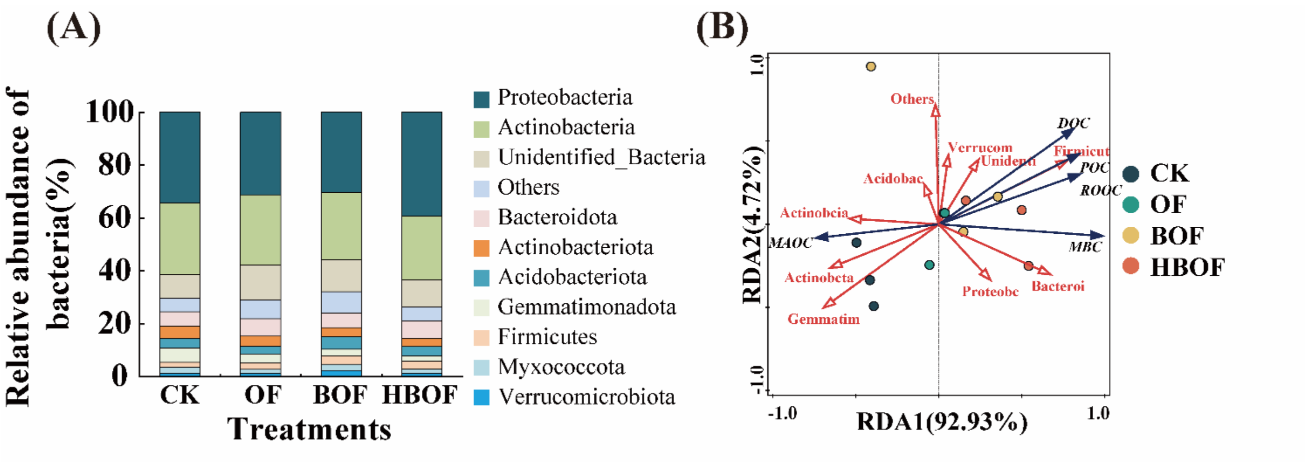

Supplement: Supplementary file 1 [file Data_Sheet_1.pdf]
